# Akt1 Stimulates Homologous Recombination Repair of DNA Double-Strand Breaks in a Rad51-Dependent Manner

**DOI:** 10.3390/ijms18112473

**Published:** 2017-11-20

**Authors:** Katharina Mueck, Simone Rebholz, Mozhgan Dehghan Harati, H. Peter Rodemann, Mahmoud Toulany

**Affiliations:** 1 Division of Radiobiology and Molecular Environmental Research, Department of Radiation Oncology, University of Tuebingen, 72076 Tuebingen, Germany; katharina.mueck@uni-tuebingen.de (K.M.); simone.rebholz@klinikum.uni-tuebingen.de (S.R.); mozhgan.dehghan-harati@klinikum.uni-tuebingen.de (M.D.H.); 2 German Cancer Consortium (DKTK), Partner site Tuebingen, and German Cancer Research Center (DKFZ), 69120 Heidelberg, Germany

**Keywords:** homologous recombination, Akt1, Rad51, DNA double-strand break repair, non-small cell lung cancer

## Abstract

Akt1 is known to promote non-homologous end-joining (NHEJ)-mediated DNA double-strand break (DSB) repair by stimulation of DNA-PKcs. In the present study, we investigated the effect of Akt1 on homologous recombination (HR)-dependent repair of radiation-induced DSBs in non-small cell lung cancer (NSCLC) cells A549 and H460. Akt1-knockdown (Akt1-KD) significantly reduced Rad51 protein level, Rad51 foci formation and its colocalization with γH2AX foci after irradiation. Moreover, Akt1-KD decreased clonogenicity after treatment with Mitomycin C and HR repair, as tested by an HR-reporter assay. Double knockdown of Akt1 and Rad51 did not lead to a further decrease in HR compared to the single knockdown of Rad51. Consequently, Akt1-KD significantly increased the number of residual DSBs after irradiation partially independent of the kinase activity of DNA-PKcs. Likewise, the number of residual BRCA1 foci, indicating unsuccessful HR events, also significantly increased in the irradiated cells after Akt1-KD. Together, the results of the study indicate that Akt1 seems to be a regulatory component in the HR repair of DSBs in a Rad51-dependent manner. Thus, based on this novel role of Akt1 in HR and the previously described role of Akt1 in NHEJ, we propose that targeting Akt1 could be an effective approach to selectively improve the killing of tumor cells by DSB-inducing cytotoxic agents, such as ionizing radiation.

## 1. Introduction

Non-small cell lung cancer (NSCLC) accounts for the largest subgroup of lung cancers, resulting in the highest portion of cancer-related mortality worldwide [1,2,3,4]. Chemo-/radiotherapy represents the standard treatment for locally advanced NSCLC. However, treatment resistance can occur, resulting in 3-year survival rates of 15–20% [5,6]. The PI3K/Akt pathway is frequently hyperactivated in NSCLC [7,8,9]. The serine/threonine kinase Akt, also known as protein kinase B (PKB), exists in three isoforms, namely, Akt1/PKBα, Akt2/PKBβ and Akt3/PKBγ [10,11]. A high level of activated Akt1 in tumor specimens is a prognostic factor for poor outcomes in NSCLC [12]. In addition to stimulating tumor cell proliferation, growth and survival [7], Akt, especially Akt1, promotes DNA double-strand break (DSB) repair and clonogenic survival after irradiation [13,14,15,16]. Various Akt inhibitors are currently being tested in clinical studies, demonstrating overall tolerable toxicities and promising anti-tumor activities [17,18,19].

DSBs are the most severe form of irradiation-induced DNA lesions, which can lead to cell death [20]. γH2AX foci are clusters of histones, which get phosphorylated adjacent to DSBs and, thus, serve as DSB markers [21,22]. Cells are capable of DSB repair, a process, which is usually completed within 24 h after DSB induction [23]. γH2AX histones strongly promote recruitment of DSB repair proteins and are crucial for efficient repair [24,25,26]. The main pathways for the repair of irradiation-induced DSBs are non-homologous end-joining (NHEJ) and homologous recombination (HR) [27,28]. Akt1 has been shown to promote NHEJ via interaction with and stimulation of DNA-dependent protein kinase catalytic subunit (DNA-PKcs) in various cell lines including NSCLC cells A549 and H460 [13,14,15,29,30,31].

HR is a major repair pathway for irradiation-induced DSBs in late S and G2 phases [32,33,34,35,36]. In addition, HR is critical for the repair of broken replication forks and DNA interstrand crosslinks [32,37]. HR-mediated repair uses a homologous DNA sequence, usually the sister chromatid, as the repair template resulting in high-fidelity repairs. The first step of HR repair is end resection of DSBs to obtain 3′ single-stranded DNA (ssDNA) overhangs. After that, the 3′ ssDNA overhang invades a homologous double-stranded DNA (dsDNA) sequence. The invading 3′ ssDNA is elongated by DNA synthesis, and the triple helix structure is resolved [38,39]. DSB end resection is facilitated by a protein complex that includes BRCA1. Invasion of the ssDNA overhang into the homologous dsDNA sequence is dependent on the recombinase Rad51. Accordingly, decreased Rad51/BRCA1 foci formation at the DSB is an indicator for impaired recruitment and decreased HR-dependent repair [40,41,42,43,44]. Conversely, an elevated number of residual Rad51/BRCA1 foci suggests reduced foci resolution and impaired HR repair [39,45].

So far, only a few studies have investigated the possible role of Akt1 in the regulation of HR-dependent DSB repair. Akt1 has been reported to impair the nuclear localization and foci formation of BRCA1/Rad51 as well as HR repair in normal tissue and breast cancer cells [46,47,48]. However, Rad51 foci formation has been demonstrated to be independent of Akt activity in HEK cells following etoposide treatment [49]. Conversely, Akt has been shown to stimulate Rad51 protein expression in several NSCLC cell lines including A549 cells [50,51,52]. Nonetheless, so far it is not known whether the promotive effect of Akt on Rad51 protein level influences HR-mediated DSB repair in NSCLC cells.

In this study, we investigated the effect of Akt1 on HR-dependent DSB repair and the possible underlying mechanism primarily in NSCLC cells A549 as well as in H460 cells. Moreover, we sought to elucidate whether the modulation of HR repair by Akt1 affects clonogenicity after irradiation. We demonstrated that Akt1 stimulates HR-mediated DSB repair in a Rad51-dependent manner. This contributes to the stimulatory effect of Akt1 on DSB repair following irradiation. Thus, our data provide further insights into the role of Akt1 in DSB repair.

## 2. Results

### 2.1. Akt1 Promotes HR-Dependent DSB Repair

**Figure 1:**
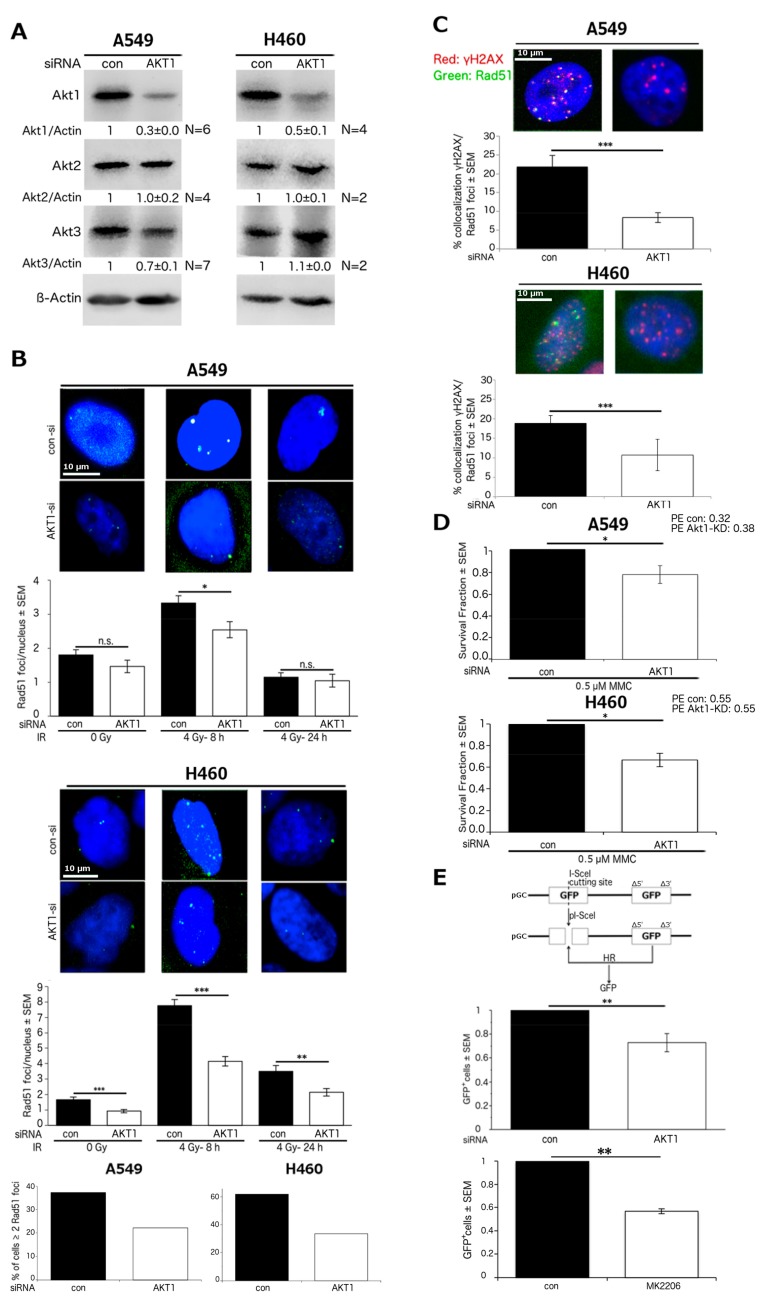
Akt1 promotes HR-dependent DSB repair. A549 and H460 cells were transfected with AKT1-siRNA or con-siRNA. (**A**) The protein levels of Akt1, Akt2 and Akt3 were analyzed by Western blotting. β-Actin was used as the loading control. The protein levels were normalized to those in the con-siRNA transfected cells. The data represent the mean ± SEM of the indicated number of independent experiments; (**B**) the Rad51 foci assay was performed as described in the Methods section at the indicated time points after irradiation. The bars represent the mean number of foci/cell ± SEM from at least 3 independent experiments. At least 276 nuclei per condition were evaluated. Bars showing the percentage of cells with at least 2 Rad51 foci/nucleus are based on data for the 8 h time point. Akt1-KD significantly decreased Rad51 foci formation (* *p* < 0.05, ** *p* < 0.01, *** *p* < 0.001, Student’s *t*-test); (**C**) 8 h after irradiation, the fraction of γH2AX foci colocalized with Rad51 foci was examined. The results are based on the mean number of foci from two independent experiments, a total of at least 175 nuclei per condition for A549 cells, and 2 independent experiments, a total of at least 197 counted nuclei for H460 cells (*p* < 0.001, Student’s *t*-test); (**D**) clonogenic survival after MMC treatment was analyzed (A549, *n* = 4, 12 data points; H460, *n* = 1, 3 data points; * *p* < 0.05, Student’s *t*-test); (**E**) HR-reporter assay was performed. A549 cells were treated with the indicated siRNAs or MK2206 (10 μM)/DMSO. The fraction of con-siRNA transfected cells, which were GFP-positive, equaled 0.56 ± 0.11%, the proportion of Akt1-KD cells equaled 0.40 ± 0.07%. The proportion of DMSO control cells, which were GFP-positive, equaled 1.52 ± 0.18%. The portion of GFP-positive MK2206 treated cells equaled 0.86 ± 0.10% (raw data). The raw values of GFP-positive cells were normalized to those in the con-siRNA/DMSO treated cells. Akt1-KD significantly reduced the fraction of GFP-positive cells (*n* = 3, 9 data points; ** *p* < 0.01, Student’s *t*-test). MK2206 treatment also significantly reduced the fraction of GFP-positive cells (*n* = 2, 3 data points; ** *p* < 0.01, Student’s *t*-test). PE: Plating Efficiency.

We analyzed the effect of Akt1 on HR in NSCLC cells using A549 and H460 cells, in which Akt1 was knocked down by siRNA. Akt1-knockdown (Akt1-KD) did not affect the protein level of Akt2, although it slightly reduced Akt3 protein expression in A549 cells but not in H460 cells (Figure 1A). The influence of Akt1-KD on Rad51 foci formation was tested in non-irradiated cells as well as in cells 8 and 24 h post irradiation with 4 Gy. These time points were chosen based on the time-course of Rad51 foci formation in A549 cells, which shows a peak between 4 and 8 h after irradiation (Figure S1). Furthermore, compared to 4 h post-irradiation, the Rad51 foci appeared brighter and more distinct at the 8 h time point. As shown in Figure 1B, irradiation significantly increased Rad51 foci number as compared to non-irradiated cells at 8 h after irradiation in A549 and H460 cells (*p* < 0.001). In A549 cells, Akt1-KD subtly, yet significantly reduced Rad51 foci number in comparison to con-siRNA transfected cells at 8 h post-irradiation. In the non-irradiated A549 cells or in cells at 24 h after irradiation, the number of Rad51 foci was not influenced by the depletion of Akt1. In H460 cells, Akt1-KD significantly reduced the number of Rad51 foci in the non-irradiated cells. Moreover, Akt1 depletion significantly decreased the number of Rad51 foci in the cells at 8 and 24 h post-irradiation. Based on the same data sets, we determined the fraction of cells with at least 2 Rad51 foci/nucleus in A549 and H460 cells 8 h after irradiation. The threshold of 2 foci was chosen based on the basal foci number in non-irradiated cells. In both cell lines, the proportion of cells with 2 Rad51 foci or more than 2 foci was reduced by about 50% after Akt1-KD. Conversely, Akt1-KD has been reported to increase BRCA1 foci formation in MCF-7 breast cancer cells at 12 h after irradiation [47]. Interestingly, we also observed that Akt1-KD significantly increases the number of radiation-induced Rad51 foci in MCF-7 cells at 12 h after irradiation (Figure S2).

To further investigate the effect of Akt1 on HR in NSCLC cells, we examined the influence of Akt1 depletion on the colocalization of γH2AX and Rad51 foci in A549 and H460 cells at 8 h after 4 Gy irradiation. As shown in Figure 1C, the proportion of γH2AX foci that were colocalized with Rad51 foci significantly decreased by more than half in A549 cells following Akt1-KD. Likewise, Akt1 depletion significantly reduced the fraction of γH2AX foci colocalized with Rad51 foci by about half in H460 cells.

Mitomycin C (MMC) treatment leads to DSBs that are primarily repaired via HR [37,53]. Therefore, we examined the influence of Akt1-KD on clonogenic survival following MMC treatment. Since colony formation assay takes 10 days, we already confirmed the stability of Akt1-KD over a period of 7 days, the time required for reaching majority of colonies to 50 cells or more [31]. As demonstrated in Figure 1D, Akt1 depletion significantly reduced the clonogenic survival of A549 and H460 cells after MMC administration.

The effect of Akt1-KD was also tested using HR-reporter assays in A549 cells. The data shown in Figure 1E and Figure S3 demonstrate that Akt1-KD significantly decreased the relative proportion of GFP-positive cells, which indicates the inhibition of HR. Furthermore, treatment with the specific Akt inhibitor MK2206 also significantly reduced HR repair in A549 cells. Raw values of GFP expressing cells are stated in the figure legend. H460 cells could not be used for the HR-reporter assay due to massive cell death after plasmid transfection. Thus, we focused on the A549 cell line for further analyses.

### 2.2. Stimulation of HR Repair by Akt1 is Dependent on Rad51

**Figure 2:**
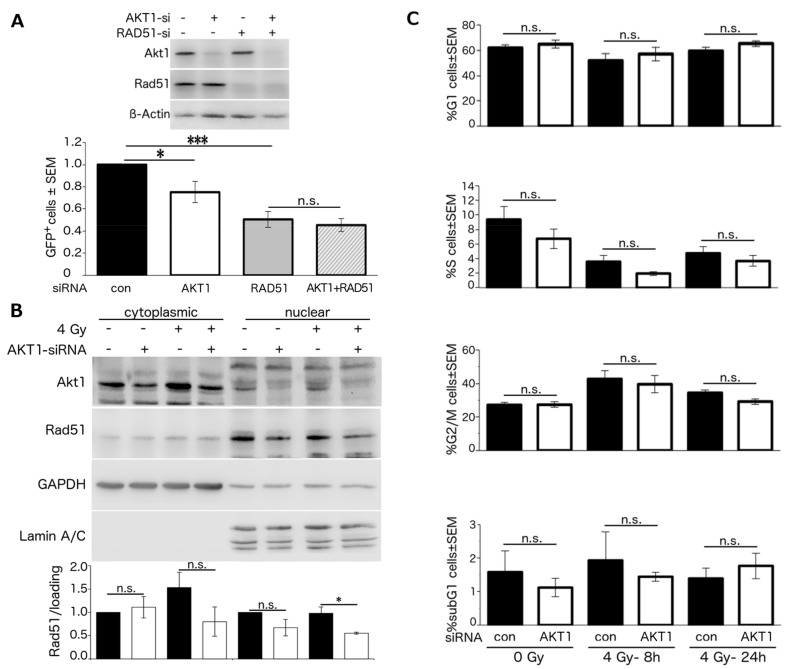
Stimulation of HR repair by Akt1 is dependent on Rad51. (**A**) HR-reporter assay was performed in A549 cells after transfection with the indicated siRNAs. The fraction of con-siRNA transfected cells, which were GFP-positive, equaled 0.46 ± 0.10%, for Akt1-KD 0.33 ± 0.05%, for Rad51-KD 0.24 ± 0.05%, for Akt1-KD+Rad51-KD 0.20 ± 0.03% (raw data). The raw values of GFP-positive cells were normalized to con-siRNA transfected cells. Asterisks indicate significant reduction of GFP-positive cells by Akt1-KD and Rad51-KD (*n* = 4, at least 8 data points; * *p* < 0.05, *** *p* < 0.001); (**B**) following transfection with AKT1-siRNA, A549 cells were irradiated, and 8 h later, the cytoplasmic and nuclear fractions were prepared. Rad51 protein levels were determined by Western blotting. GAPDH and Lamin A/C were used as cytoplasmic and nuclear markers, respectively. Densitometry is based on the mean ± SEM of 3 independent experiments. Akt1-KD significantly reduced Rad51 protein level (* *p* < 0.05); (**C**) A549 cells were treated with AKT1-siRNA, harvested at the indicated time points post irradiation, and cell cycle distribution was examined (*n* = 3, 6 data points). n.s., not significant.

To test whether Akt1-KD impairs HR repair through the inhibition of Rad51, we performed HR-reporter assays in A549 cells after single and double knockdown of Akt1 and Rad51. The depletion of Akt1 or Rad51 alone significantly inhibited HR as shown by reduced GFP expression. Likewise, the relative proportion of GFP-positive cells was significantly decreased after the concurrent depletion of Akt1 and Rad51. However, no further inhibition of HR was achieved after double knockdown of Akt1 and Rad51 compared to the single knockdown of Rad51. Raw values of GFP expressing cells are given in the figure legend (Figure 2A and Figure S4).

Next, we examined the effect of Akt1-KD on Rad51 protein levels in the cytoplasmic and nuclear fractions of non-irradiated A549 cells and in cells 8 h post 4 Gy irradiation. Knockdown of Akt1 did not affect the Rad51 protein level in the cytoplasmic fraction of non-irradiated cells. However, the amount of Rad51 protein slightly decreased in the nuclear fraction of non-irradiated cells and in the cytoplasmic fraction of irradiated cells following Akt1-KD. Importantly, Akt1 depletion significantly reduced the amount of Rad51 protein in the nuclear fraction following irradiation (Figure 2B).

It is known that HR-mediated repair is active in late S phase and G2 phase [32,33,34,35,36]. We analyzed the cell cycle distribution in non-irradiated A549 cells as well as in cells 8 and 24 h after 4 Gy irradiation following Akt1-KD. As shown in Figure 2C, Akt1 depletion did not significantly affect the proportion of cells in the different phases of the cell cycle.

### 2.3. Akt1 Promotes DSB Repair after Irradiation Partially by Stimulation of HR Repair

**Figure 3:**
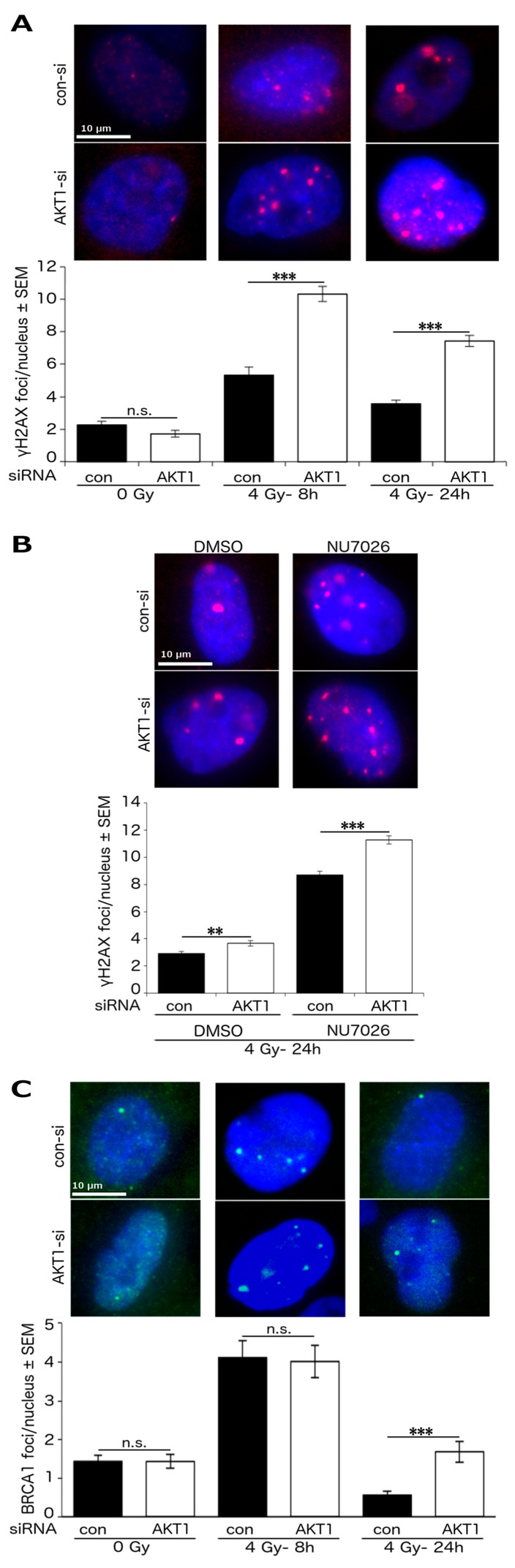
Akt1 promotes DSB repair after irradiation partially by stimulation of HR repair. A549 cells were transfected with AKT1-siRNA or con-siRNA. (**A**) The number of γH2AX foci was analyzed at the indicated time points post irradiation. The results at 8 h after irradiation represent the mean ± SEM of 2 independent experiments and a total of at least 158 evaluated nuclei per condition. The data for the non-irradiated cells and the cells at 24 h post irradiation are based on the mean ± SEM of 3 independent experiments and a total of at least 275 nuclei per condition. The number of γH2AX foci/nucleus was significantly increased after Akt1-KD (*** *p* < 0.001, Student’s *t*-test); (**B**) following treatment with the DNA-PKcs inhibitor NU7026 (10 μM) and irradiation, γH2AX foci assay was performed. The data represent the mean ± SEM of 3 independent experiments and a total of at least 320 evaluated nuclei per condition. Akt1-KD significantly increased the number of residual γH2AX foci/nucleus (** *p* < 0.01, *** *p* < 0.001, Student’s *t*-test); (**C**) the number of BRCA1 foci was determined at the indicated time points after irradiation. The results are based on the mean ± SEM of 3 independent experiments and a total of at least 287 nuclei per condition. Asterisks indicate a significant increase in the number of residual BRCA1 foci/nucleus following Akt1-KD (*** *p* < 0.001, Student’s *t*-test).

We analyzed the effect of Akt1-KD on γH2AX foci formation in A549 cells after single dose irradiation with 4 Gy (after 8 and 24 h). Akt1-KD significantly increased the number of γH2AX foci at 8 and 24 h following irradiation (Figure 3A).

It is known that Akt1 stimulates NHEJ-mediated repair by interacting with DNA-PKcs [14,15,29]. Therefore, as a parameter of DSB repair efficacy, we examined the number of residual γH2AX foci in A549-Akt1-KD cells 24 h after 4 Gy irradiation and concurrent inhibition of DNA-PKcs by NU7026. Akt1 depletion alone significantly increased the number of residual γH2AX foci. Moreover, the number of γH2AX foci significantly increased after NU7026 treatment when the A549-Akt1-KD cells were compared to the non-transfected A549 cells (Figure 3B).

Furthermore, the comparison of the number of BRCA1 foci revealed that knockdown of Akt1 did not alter the formation of BRCA1 foci in the irradiated or non-irradiated cells. However, we observed that the number of residual BRCA1 foci 24 h post-irradiation, indicative of unsuccessful HR events, significantly increased in the Akt1-KD cells (Figure 3C).

### 2.4. Akt1-Mediated HR Repair Plays a Minor Role in Post-Irradiation Clonogenic Survival

HR repair is available in late S phase and G2 phase of the cell cycle [32,33,34,35,36]. To elucidate the contribution of HR repair on the Akt1-mediated improvement in post-irradiation clonogenic survival, we used non-synchronized and S/G2 phase synchronized cells. The synchronization of cells in S/G2 phase by aphidicolin led to a ~2-fold increase in the proportion of cells in S phase and a marked increase in the proportion of cells in G2/M phase (Table 1).

**Figure 4:**
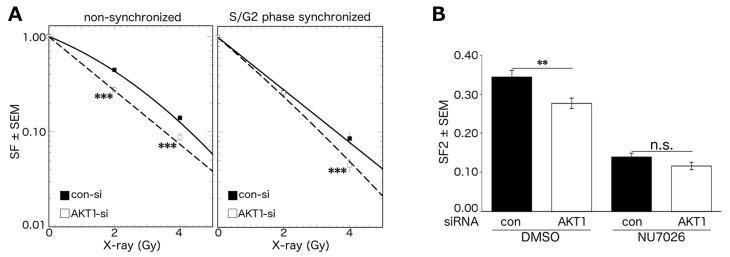
Akt1 increases post-irradiation clonogenic survival via NHEJ repair. A549 cells were treated with AKT1-siRNA or con-siRNA. (**A**) Cells were synchronized in S/G2 phase or kept non-synchronized. After irradiation with the indicated doses, clonogenic assays were performed by immediate plating (PE non-synch/con: 0.35, PE non-synch/Akt1-KD: 0.32, PE synch/con 0.16, PE synch/Akt1-KD: 0.23). Akt1-KD significantly decreased the post-irradiation clonogenic survival (*n* = 3, 36 data points; *** *p* < 0.001, Student’s *t*-test); (**B**) Cells were treated with the DNA-PKcs inhibitor NU7026 (10 μM) and irradiated with 2 Gy. Six hours after irradiation, clonogenic assays were performed by delayed plating (PE DMSO/con: 0.26, PE DMSO/Akt1-KD: 0.29, PE NU7026/con 0.25, PE NU7026/Akt1-KD: 0.25). Asterisks indicate significant reduction in clonogenic survival after Akt1-KD (*n* = 3, 9 data points; ** *p* < 0.01, Student’s *t*-test). SF: surviving fraction; SF2: surviving fraction after irradiation with 2 Gy; PE: Plating Efficiency.

The non-synchronized Akt1-KD cells displayed a significantly reduced post-irradiation clonogenic survival when compared to the control cells (D_37_Control: 2.3 ± 0.3 vs. D_37_Akt1-KD: 1.8 ± 0.1). However, the post-irradiation clonogenic survival of log-phase S/G2 synchronized cells was only slightly reduced following Akt1-KD (D_37_Control: 1.5 ± 0.1 vs. D_37_Akt1-KD: 1.4 ± 0.1) (Figure 4A). In addition, we examined the post-irradiation clonogenicity of A549 cells after Akt1-KD and concurrent DNA-PKcs inhibition by NU7026. As shown in Figure 4B, Akt1 depletion alone significantly reduced the clonogenic fraction after irradiation. However, Akt1-KD did not reduce the clonogenic survival in cells following DNA-PKcs inhibition.

## 3. Discussion

The data from the Akt1-KD experiments presented here indicate that Akt1 plays a regulatory role not only for NHEJ repair, as has been reported earlier [14,15,29,30], but also for HR repair in a Rad51-dependent manner.

To this aim, the results of Rad51 protein expression and foci formation together with the data from the HR-reporter assays strongly suggest that Akt1 stimulates HR-dependent DSB repair. The overall higher number of radiation-induced Rad51 foci in H460 cells as compared to A549 cells is in line with an earlier report describing cell line-dependent differences in the extent of radiation-induced Rad51 foci formation [54]. Both, A549 and H460 cells are EGFR and TP53 wildtype, but harbor an activating KRAS mutation. In contrast to A549 cells, the H460 cell line shows an activating mutation of PIK3CA coding for PI3K, which acts upstream of Akt1 [55,56]. This might underlie the enhanced phosphorylation level of Akt1 (S473, T308) in H460 cells as compared to A549 cells, which has been reported previously [57]. The higher phosphorylation level, i.e., Akt1 activation, might explain the herein observed more pronounced effect of Akt1-KD on Rad51 foci number in H460 cells compared to A549 cells. Furthermore, Rad51 foci formation is influenced by several other factors such as recruitment of RPA and BRCA2 as well as other repair proteins like Rad52 [58,59]. It is possible that these factors are differentially activated in both cell lines, which might also account for the different extent of reduced Rad51 foci number in A549 and H460 cells after Akt1-KD. Another possible explanation might be the strongly increased amount of γH2AX foci in A549-Akt1-KD cells at 8 h after irradiation. In contrast, we reported previously [31] that in H460 cells Akt1 depletion significantly enhances the number of residual γH2AX foci 24 h after irradiation, but does not affect the number of γH2AX foci at the time point 8 h post-irradiation. It is well known that H2AX phosphorylation is crucial for the recruitment of DSB repair proteins like Rad51 to the damage site [24,25,26]. Thus, it seems plausible that the increased number of γH2AX foci promotes recruitment of Rad51 to the damage site in A549-Akt1-KD cells, while at the same time Akt1-KD reduces Rad51 protein level and consequently foci formation. This interpretation is supported by the observation that Akt1 depletion decreased the fraction of γH2AX foci, colocalized with Rad51 foci, by more than half in A549 cells and approximately half in H460 cells. Contrary to individual data for γH2AX foci and Rad51 foci, looking at colocalization of γH2AX with Rad51 foci allows adjusting for the above-mentioned stimulatory effect of γH2AX foci on recruitment of Rad51. The reason why Akt1-KD affected the number of γH2AX foci more strongly in A549 cells than H460 cells at 8 h after irradiation is speculative, but might indicate that Akt1 exerts a more pronounced effect on early DSB repair in A549 cells compared to H460 cells.

In addition to foci experiments, the reduced clonogenic survival after MMC treatment further indicates that Akt1 promotes HR in A549 and H460 cells. We already tested the stability of Akt1-KD over a period of 7 d in a previous publication showing excellent stability [31]. Evaluation of Akt1-KD by Western blotting over a period of 10 d (in accordance with duration of clonogenic assay) was not possible due to technical reasons, i.e., cells could not be grown in a monolayer without media change for 10 days. Anyhow, knockdown stability during the first days of clonogenic assay is most important, since the first 24 h after DNA damage induction are most crucial for repair of DSBs, which manifests after several days as clonogenic cell death [21,23]. DSBs, which result from MMC treatment, are primarily and accurately repaired via the HR repair pathway [37]. Misrepair of MMC-mediated DSBs by NHEJ increases MMC-induced cytotoxicity. Consequently, inhibition of the NHEJ pathway has been shown to partially rescue clonogenicity after MMC treatment [37,60]. Thus, the observed modest Akt1-KD-mediated sensitization to MMC is in accordance with the stimulatory role of Akt1 in both HR- and NHEJ-dependent repair.

The stimulation of HR repair by Akt1 seems to be in contrast to reports by Plo et al. [47,48] and Jia et al. [46]. These authors have reported an inhibitory effect of Akt1 overexpression on Rad51 foci formation and HR repair using an HR-reporter assay in normal tissue and breast cancer cells [46,47,48]. In line with this report, we observed an increase in the number of Rad51 foci in the breast cancer cell line MCF-7 after Akt1-KD. On the other hand, the data using the two NSCLC cell lines (A549 and H460) presented here are in accordance with the report by Ko et al., who demonstrated that Akt inhibition reduced the clonogenicity of NSCLC cells A549 and H1703 after MMC treatment [51]. The differential effect of Akt1 reported in earlier studies [46,47,48] and our current study might indicate a cell line-dependent role of Akt1 in HR.

With respect to the mechanism underlying the stimulatory function of Akt1 in HR repair, our data indicate that Akt1 increases Rad51 protein level, especially in the nucleus after irradiation and as a result enhances Rad51 foci formation at the DSB. Lack of a significant difference in HR repair between the cells with single knockdown of Rad51 and those with Akt1/Rad51 double knockdown implies that Akt1 reduces HR repair in a Rad51-dependent manner. These results are supported by results from other studies reporting that inhibition of Akt reduces Rad51 protein levels in different NSCLC cell lines including A549 cells [50,51,52,61]. Moreover, Ko et al. demonstrated that sensitization of NSCLC cells to MMC after Akt1 inhibition is partially rescued by Rad51 overexpression [51]. This implies that Akt1 inhibition, which is known to reduce NHEJ [14,15,29,30], also impairs HR in a direct manner but does not lead to a passive switch from NHEJ to HR in NSCLC cells. Even though Rad51 protein level [62,63,64] and HR repair capacity [32,33,34,35,36] are known to be highest during S/G2 phase, interference with the cell cycle does not seem to underlie the effect of Akt1 on Rad51, since cycle distribution of Akt1-KD cells was not significantly affected. In contrast, earlier reports suggest that Akt increases Rad51 protein level by stimulating Rad51 mRNA and protein stability via reduction of Rad51 ubiquitination and proteasomal degradation [50,51].

The data from the γH2AX and BRCA1 foci assays presented here support the conclusion that Akt1 not only promotes NHEJ-mediated repair of radiation-induced DSBs but also exerts a stimulatory role on HR repair. The increased number of γH2AX foci represents non-repaired DSBs [21,22], most likely a consequence of both impaired HR- and NHEJ-mediated DSB repair after Akt1-KD. However, Akt1-KD led to an even more pronounced increase in the number of γH2AX foci in A549 cells when it was combined with a DNA-PKcs inhibitor in comparison with that in cells with unhampered NHEJ. Hence, the increase in the number of γH2AX foci after DNA-PKcs inhibition plus Akt1-KD is at least partly due to the inhibition of DSB repair independent of the effect of Akt1 on DNA-PKcs activity, in other words, it is most likely due to the influence of Akt1 on HR. The number of residual Rad51/BRCA1 foci (24 h after irradiation) is a common indicator for the removal of the repair protein after successful repair and thus forms a marker of non-repaired DSBs as a result of hampered HR repair [39,45]. In contrast, the number of Rad51/BRCA1 foci at closer time points after DSB induction (~4–12 h after irradiation) indicates the assembly of the protein at the DSB [39,47]. Thus, our results suggest that Akt1 stimulates the recruitment of Rad51 to the damage site, whereas BRCA1 recruitment does not seem to be affected. As a consequence of decreased Rad51 protein assembly at the DSB, HR repair cannot be completed and the BRCA1 protein, which was recruited to the damage before Rad51, cannot be removed from the damage leading to the increased number of residual BRCA1 foci. Thus, this further supports the hypothesis that Akt1 enhances repair of radiation-induced DSBs partly by HR. Interestingly, we observed that the Akt1-KD-mediated impairment of DSB repair was weaker after treatment with DMSO as solvent control in A549 cells. The reason for this is speculative but might be attributed to the stimulatory effect of DMSO on NHEJ [65]. Nonetheless, the data presented here are in accordance with several studies, showing impaired DSB repair after irradiation following AKT1-siRNA or the pharmacological inhibition of Akt [13,14,15,16]. However, in a previous report, Akt1 depletion did not increase the number of residual γH2AX foci in A549 cells when NHEJ was impaired by DNA-PKcs-KD [14]. Yet, this experiment was carried out using cells in stationary phase, whereas we used cells in log-phase for the present study. Accordingly, our result is in line with the role of the HR pathway in DSB repair in late S and G2 phases, but not in the G1 phase.

Conversely, the data from colony formation assays indicate that HR plays a minor role in the Akt1-mediated stimulation of clonogenic survival after irradiation. The reduction in clonogenic survival after Akt1-KD and irradiation is in line with previous studies, showing radiosensitization by Akt inhibition or AKT1-siRNA [14,15,16]. The strong radiosensitization of non-synchronized cells but only slight radiosensitization of S/G2 phase synchronized cells following Akt1-KD fits the pattern of NHEJ-deficient but not HR-deficient cells [66]. Furthermore, the lack of effect of Akt1-KD on clonogenicity after DNA-PKcs inhibition and irradiation is supported by a previous study, showing that Akt1-KD does not induce radiosensitization in DNA-PKcs-deficient cells [15]. Thus, our results imply that Akt1-KD-mediated reduction of HR decreases clonogenic survival after MMC treatment but does not affect clonogenicity after irradiation. This finding is supported by previous studies, which show that impairment of HR increases sensitivity to MMC to a greater degree than to irradiation [67,68]. It is known that HR is a predominant factor for survival after MMC [37,53], whereas clonogenic survival after irradiation is influenced by several DNA repair pathways [69,70,71] and additional factors such as autophagy [72,73,74] which might shadow the effect of reduced HR on post-irradiation clonogenicity. Nonetheless, the reason behind why decreased HR repair after Akt1-KD reduces DSB repair following irradiation but does not affect clonogenic survival is speculative. The lack of correlation between DSB repair and clonogenic survival might be explained by other influences, such as autophagy. Several publications have shown that autophagy increases radioresistance [72,73,74]. Moreover, Akt1-KD as well as the inhibition of HR proteins such as BRCA1 is known to induce autophagy and subsequently, increase tumor cell survival [75,76,77,78]. Thus, Akt1-KD-mediated HR inhibition might lead to enhanced autophagy, which could be sufficient to counteract the radiosensitizing effect of Akt1-KD through impaired HR-mediated DSB repair.

In conclusion, Akt1 promotes HR repair in a Rad51-dependent manner in NSCLC cells A549 and H460. Thus, our data provide further insights into the Akt-mediated resistance of NSCLC to chemo- and radiotherapy. Although further analyses are necessary to investigate the functional interaction between Akt1 and Rad51 in stimulating DSB repair, the present study offers new aspects for the development of novel strategies for selective targeting of NSCLC cells.

## 4. Materials and Methods

### 4.1. Cell Culture

A549 cells (ATCC^®^ CCL-185™) and MCF-7 (ATCC^®^ HTB-22™) were grown in Dulbecco’s Modified Eagle’s Medium (DMEM, Thermo Fisher Scientific, Darmstadt, Germany). H460 (ATCC^®^ HTB-177™) cells were cultured in RPMI medium (Thermo Fisher Scientific). Media were supplemented with 10% fetal calf serum and 1% penicillin/streptomycin in a humidified atmosphere of 93% air/7% CO_2_ at 37° C. All experiments were carried out using log-phase cells at the time of irradiation.

### 4.2. Antibodies and Reagents

The anti-Akt1 antibody (Cat#610877) was a product of BD Biosciences (Heidelberg, Germany). The antibodies against Akt2 (Cat#2964), Akt3 (Cat#8018), BRCA1 (Cat#9010) and GAPDH (Cat#2118) were obtained from Cell Signaling (Frankfurt, Germany). The anti-β-Actin antibody (Cat#A2066) was purchased from Sigma-Aldrich (Taufkirchen, Germany). The antibodies against Lamin A/C (Cat#40567) and Rad51 (Cat#88572) were provided by abcam (Cambridge, UK). The anti-P-H2AX (S139) antibody (Cat#05-636) was a product of Merck Millipore (Darmstadt, Germany). Alexa Fluor 488 goat anti-mouse (Cat#A11001) and goat anti-rabbit antibodies (Cat#A11008) as well as Lipofectamine 2000 (Cat#11668027) were purchased from Thermo Fisher Scientific. The Cy3 donkey anti-rabbit antibody (Cat#711-165-152) was a product of Jackson ImmunoResearch (Suffolk, UK). The siRNAs against Akt1 (Cat#M-003000-03-0005) and Rad51 (Cat#L-003530-00-0005) as well as the non-targeting siRNA (Cat#D-001810-10-20) were purchased from Dharmacon (Bonn, Germany), USA). Mitomycin C (approval#394.01.00) was obtained from Medac (Wedel, Germany). The DNA-PKcs inhibitor NU7026 (Cat#S2893) and the Akt inhibitor MK2206 (Cat#S1078) were purchased from Selleckchem (Munich, Germany). The plasmids pGC and pI-SceI were provided by Dr. Wael Mansour (Laboratory of Radiobiology & Experimental Radiooncology, University Medical Center Hamburg-Eppendorf, Germany). Development of plasmids pGC and pI-SceI has been described previously [27].

### 4.3. siRNA Transfection

Cells were transiently transfected with 50 nM of a pool of 4 different siRNAs against Akt1 and/or 25 nM of a pool of 4 different siRNAs against Rad51 or an equal concentration of the non-targeting siRNA using Lipofectamine 2000. Whole cell lysates were prepared 48 h and 72 h after transfection to analyze the knockdown efficiency by Western blotting.

### 4.4. Rad51, γH2AX and BRCA1 Foci Assays

Two days after siRNA transfection, cells were irradiated with 4 Gy. For the inhibition of DNA-PKcs, the cells were treated with the DNA-PKcs inhibitor NU7026 (10 μM) or DMSO 2 h before irradiation. At 8 or 24 h post-irradiation, Rad51, γH2AX and BRCA1 foci assays were performed as described previously [13]. The primary antibodies were used at a concentration of 1:200 for Rad51 and 1:300 for P-H2AX (S139) as well as BRCA1. We included all cells for our analysis independent of their cycle phase in order to reflect the contribution of HR on overall repair capacity in a tumor where cells are in different cell cycle phases.

### 4.5. HR-Reporter Assay

Cells were treated with siRNA and 24 h later transiently transfected with 1 μg plasmid pGC and 1 µg plasmid pI-SceI using Lipofectamine 2000. In case of MK2206 treatment, cells were treated with 10 µM of the inhibitor or an equal volume of DMSO 24 h after plasmid transfection. The plasmids were not integrated into the genome. Plasmid pGC contains two nonfunctional green fluorescent protein (GFP) coding sequences. A recognition site for the endonuclease I-Scel disrupts one of the GFP sequences. The other GFP sequence carries deletions at the ends. Transfection with plasmid pI-Scel induces a DSB at the I-SecI recognition site. This DSB is repaired by HR using the uncut GFP sequence as template, thereby producing a functional GFP coding sequence (Figure 1C). Forty-eight hours after plasmid transfection, the cells were harvested and the percentage of GFP-positive cells was analyzed by flow cytometry (FACSCalibur, BD Biosciences).

### 4.6. Subcellular Fractionation and Western Blotting

Two days after transfection with the indicated siRNA, cells were irradiated with a single dose of 4 Gy. After 8 h, subcellular fractions were prepared as described earlier [79,80]. Preparation of whole cell lysates and Western blotting were conducted as described previously [13]. Densitometry was performed using software Image Studio Light Ver 5.2 (LI-COR Biosciences, Homburg, Germany).

### 4.7. Cell Cycle Analysis

Two days after siRNA transfection, cells were irradiated with a single dose of 4 Gy. Eight and twenty-four hours after irradiation, the attached cells were harvested by trypsinization in addition to the floating cells in the media, and the cells were centrifuged and fixed in 70% ethanol. After washing with phosphate-buffered saline (PBS), the cells were incubated with RNase (100 μg/mL in PBS) for 10 min. Then, the cells were washed with PBS and stained with propidium iodide (10 μg/mL in PBS). Cell cycle distribution was analyzed by flow cytometry.

### 4.8. Synchronization of Cells in S/G2 Phase

Twenty-four hours after siRNA transfection, cells were treated with 5 μg/mL aphidicolin or DMSO for 16 h. Subsequently, the cells were washed with PBS, and fresh media was added to allow the cells to proceed to S and G2 phases. Ten hours after media change, the cells were irradiated and subjected to cell cycle analysis or colony formation assay.

### 4.9. Colony Formation Assay

Cells were treated with siRNA, synchronized in S/G2 phase or kept non-synchronized. For DNA-PKcs inhibition, the cells were treated with the DNA-PKcs inhibitor NU7026 (10 μM) or DMSO 2 h before irradiation. After irradiation with single doses of 0–4 Gy, cells were immediately plated into 6-well plates at a density of 250 cells/well for clonogenic assay. For delayed plating, cells were plated into 10 cm-dishes at a density of 1000 cells/dish 6 h after irradiation. To analyze clonogenicity after MMC administration, cells were treated with MMC (0.5 µM) 2 days after siRNA transfection. Three hours after MMC application, the cells were washed thrice with PBS and plated into 10 cm-dishes at a density of 1000 cells/dish. The cells were incubated for 10 days to allow colony formation. After staining with crystal violet, colonies of at least 50 cells were scored as survivors. The survival fractions were calculated by normalizing the plating efficiency of the irradiated/MMC-treated cells to the plating efficiency of the non-irradiated/untreated cells.

### 4.10. Statistics

Unequal Student’s *t*-test was performed to compare the data between groups. *p*-values less than 0.05 were used to define significant difference.

## Figures and Tables

**Table 1:** Cell cycle distribution after S/G2 phase synchronization. A549 cells were treated with the indicated siRNA and synchronized in S/G2 phase using aphidicolin (5 μg/mL). In parallel to the clonogenic assays, the cell cycle distribution was determined by flow cytometry (*n* = 3, at least 8 data points).

% of Cells ± SEM	Con-si		Akt1-si	
	Non-Synch.	S/G2-Synch	Non-Synch.	S/G2-Synch
G1	53.7 ± 1.3	20.9 ± 0.4	56.3 ± 0.8	33.6 ± 1.9
S	11.2 ± 0.6	23.0 ± 1.6	8.1 ± 0.6	16.7 ± 1.2
G2	32.3 ± 1.4	52.1 ± 2.1	33.9 ± 0.8	47.3 ± 2.7
subG1	2.7 ± 0.9	4.0 ± 1.0	1.7 ± 0.2	2.5 ± 0.3

## Supplementary Materials

The following are available online at www.mdpi.com/1422-0067/18/11/2473/s1.

## Abbreviations

DNA-PKcs	DNA-dependent protein kinase catalytic subunit
DSB	double-strand break
dsDNA	double-stranded DNA
GFP	green fluorescent protein
HR	homologous recombination
KD	knockdown
MMC	Mitomycin C
NHEJ	non-homologous end-joining
NSCLC	non-small cell lung cancer
PKB	protein kinase B
siRNA	short-interfering RNA
SF	surviving fraction
ssDNA	single-stranded DNA
