# Supplementary material for: Akt1 Stimulates Homologous Recombination Repair of DNA Double-Strand Breaks in a Rad51-Dependent Manner
Source: Int J Mol Sci. 2017 Nov 20;18(11):2473. doi: 10.3390/ijms18112473 (PMC5713439; doi:10.3390/ijms18112473)
Supplement: Supplementary file 1 [file ijms-18-02473-s001.pdf]

International Journal of
Molecular Sciences

Supplementary Materials: Akt1 Stimulates Homologous Recombination Repair of DNA Double-Strand Breaks in a Rad51-Dependent Manner

Katharina Mueck, Simone Rebholz, Mozhgan Dehghan Harati, H. Peter Rodemann and Mahmoud Toulany

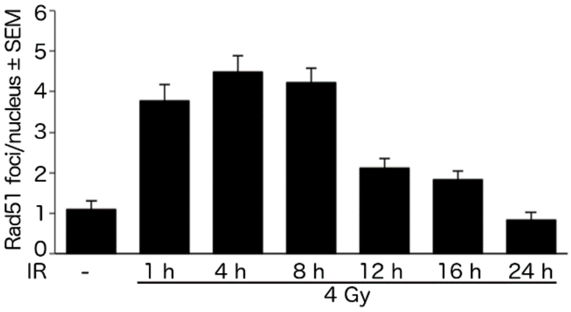

Figure. S1. Time-course of Rad51 foci formation after irradiation. Rad51 foci number was determined at the indicated time-points after irradiation with 4 Gy. (A549; N = 2, at least 163 counted nuclei/condition).

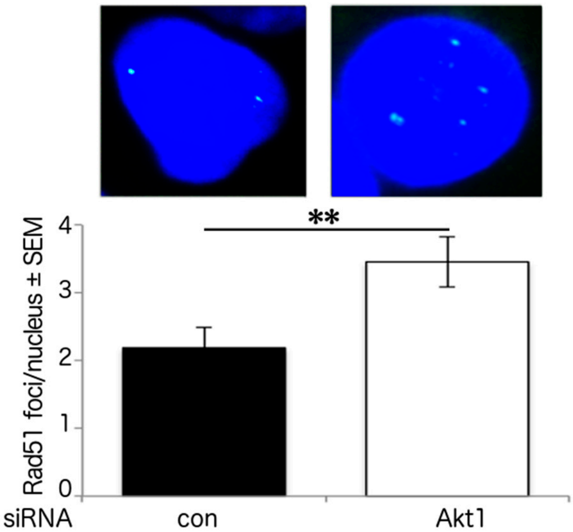

Figure S2. Effect of Akt1 on Rad51 foci formation in MCF-7 cells. Cells were transfected with AKT1- or con-siRNA. Rad51 foci number was determined 12 h after irradiation. Akt1-KD significantly increased the amount of Rad51 foci/nucleus (N = 2, at least 226 counted nuclei/condition; ** $p < 0.01$, student's *t*-test).

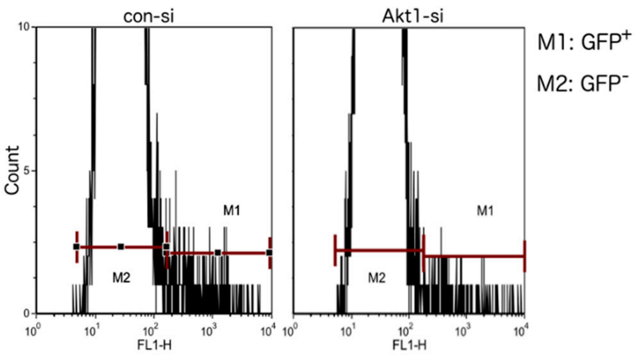

Figure S3. HR-reporter assay after Akt1-KD. Exemplary plot of GFP-expressing A549 cells following transfection with Akt1- or con-siRNA.

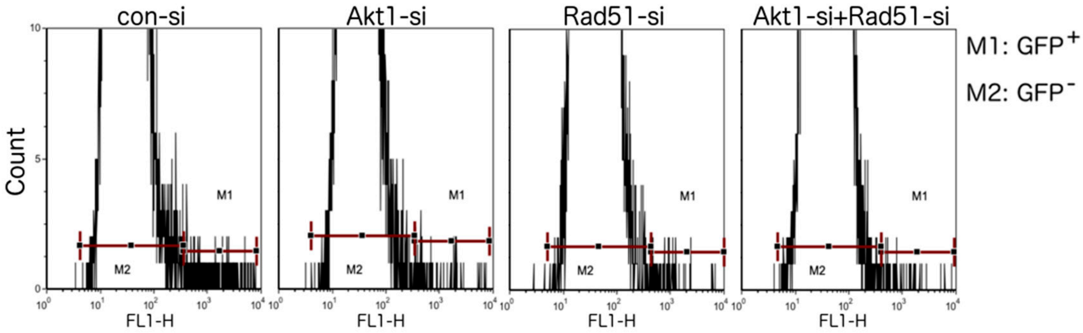

Figure S4. Effect of Akt1-KD and Rad51-KD on HR repair. HR-reporter assay was performed in A549 cells after single or concurrent knockdown of Akt1 and Rad51. Exemplary plots of GFP expression are shown.
